# Funding sources and breast cancer research frame

**DOI:** 10.1371/journal.pone.0238026

**Published:** 2020-08-24

**Authors:** Wonkwang Jo

**Affiliations:** 1 Department of Sociology, College of Social Science, Seoul National University, Seoul, Republic of Korea; 2 The Institute for Social Development and Policy Research, Seoul National University, Seoul, Republic of Korea; Fayoum University Faculty of Computers and Information, EGYPT

## Abstract

This study aims to analyze the formation of the frame of breast cancer research. To test our hypothesis that the research frame depends on the funding sources, we collected the abstracts of 48,448 breast cancer research papers from PubMed and applied structural topic modeling, word network analysis, and LASSO logistic regression to the data. In particular, we analyzed the relationship between funding sources and the molecularization of breast cancer knowledge. The results show that government-funded research is likely to have molecular objects or population as the unit of interest, whereas the research not funded by the government is likely to have individual patients as the unit of interest in relation to specific treatments. This phenomenon is attributed to the different interests of government institutions and the private sector. This study improves our understanding of molecularization and medical knowledge production.

## Introduction

The object of this study is to analyze the relationship between funding sources and the framing of breast cancer research. It is well known that medical research is not entirely independent and is exposed to social influence, at least in part [1]. Various social variables that affect medical research have been studied before. Of these, the effect of the funding source is one of the most reviewed, as funding is the social variable that most directly affects the medical research process.

Previous studies that explored the relationship between funding sources and scientific research have focused mainly on the research results. For example, many scholars have investigated whether the research receiving funding produces results that are favorable to the funders [2–4]. However, few works have examined whether the funding source is related to the *research frame* itself. The relative neglect of the relationship between funding sources and the research frame is problematic because the research frame determines which topics are studied in the first place.

Although research on the relationship between funding sources and research framing is relatively lacking, the functions and roles of framing have received much scholarly attention. Entman describes framing as “[selecting] some aspects of a perceived reality and [making] them more salient in a communicating text” [5]. According to Benford and Snow, the main tasks of framing include, among others, identifying problems and presenting solutions [6]. In short, framing amounts to separating and engaging with a specific aspect of a phenomenon. Every scientific study, insofar as it highlights an aspect of a phenomenon as a research puzzle and finds an answer to it, has a research frame.

As is well demonstrated by the study of Tversky and Kahneman, when certain framing exists, it greatly affects people's judgment and perception [7, 8]. The same is true of research frames. Although a research frame does not refer to specific opinions or content, it can induce people to focus on specific aspects of a phenomenon. For example, if a breast cancer research paper has a frame that pays attention to a cancer susceptibility gene mutation, it can lead the reader to overlook other factors (e.g., behavior and food) and focus only on gene mutation.

If funding sources are related to medical research frames in a specific direction, then certain areas would get more emphasis over others in funded studies and influence consumers of the research. This influence can exist despite conducting the study in a strictly scientific and objective manner. This effect is produced not because funding sources force specific opinion and result upon researchers, but because they impose specific topics on researchers.

This study analyzes the relationship between funding sources and the framing of breast cancer research. There are two reasons why we focus on breast cancer among many diseases. First, breast cancer research often has a powerful social influence. As one of the leading causes of death worldwide [9], cancer naturally receives a lot of social attention; accordingly, consumers of cancer research are not limited to healthcare professionals but include the wider public as well. In addition, breast cancer is one of the most common types of cancer. For example, from 2012 to 2016, breast cancer had the highest incidence rate in the United States [10]. Naturally, breast cancer and research on it are of great concern to the public and healthcare professionals alike. Second, lots of funding flows into breast cancer research. For example, from 2007 to 2016, the National Cancer Institute (NCI) devoted the highest amount of funding to research on breast cancer among all types of cancer (Based on the fiscal year. Source: The Cancer Funding Statistics of the National Cancer Institute. https://fundedresearch.cancer.gov/nciportfolio/stats.jsp). Thus, the large amount of data available on breast cancer research funding allows us to investigate the relationship between funding and medical research framing in a more rigorous manner than would be otherwise. In short, we believe that high social interest and abundant funding make breast cancer research an especially important area in investigating the dynamics of research framing.

More specifically, we focus on one of the most prevalent framings in medical research—namely, molecularization—the understanding of diseases and their treatment through molecular-level factors (e.g., genes, organelle, and hormone receptors), rather than other factors (e.g., a patient’s behavior and environment). It is well known that molecularization affects people’s perceptions and decisions [11–15]. For example, public attention to a prophylactic mastectomy cannot be explained without considering an increase in the number of studies revealing the breast cancer susceptibility genes (e.g., BRCA 1 and 2) and their effects.

Molecularization in cancer research is a social process. The results of the studies of Doll and International Agency for Research on Cancer (IARC) have shown that cancers can have numerous causes (e.g., environment, food consumption, alcohol, occupation, and radiation) [16]. This means that there are various ways to prevent or treat cancer [17, 18]. To understand why there has been a trend toward molecularization despite the various causes behind cancer, we need to examine which organization or actors are behind the phenomenon. To that end, this study attempts to reveal the relationship between different funding sources and molecularization.

To analyze the relationship between funding sources and the research frame, we used text mining methods that “take large amounts of unstructured language and quickly extract useful and novel insights” [19]. There were various attempts to measure and investigate medical framing or discourse. However, much of the existing research relies on a limited amount of data [20–22]. Moreover, although there have been studies based on larger data [23–26], they are exposed to potential threats to inference arising from the researchers’ subjective bias, because these studies often heavily rely on “Interpretivist text analysis, in which the researcher draws insights from a holistic deep reading of text” [27]. This study employs quantitative text mining methods to overcome the methodological limitations of previous research and study the relationship between social factors and scientific research in a more objective and reproducible way. Specifically, this research applies topic modeling, network analysis, and LASSO logistic regression to the abstracts of 48,448 breast cancer research papers from 1975 to 2016 and information on their funding sources taken from PubMed.

We pay particular attention to whether the funding source is the government. Since a government must manage its entire population, rather than particular individuals, they are bound to have different motives and emphases compared to individual healthcare professionals. We analyze whether this difference gets reflected in research frames via funding. As each country has a distinct research climate and thus a distinct academic world, to control for the potential effects of cross-national differences on research frames, we limit the scope of our research to studies published in the United States. The reason for choosing the United States is that the amount of medical research support there is larger than any other country in the world. According to Moses and others, as of 2011, U.S. medical research support in the public and industrial sectors accounted for 49% and 41% of global public and industrial sectors’ support, respectively [28]. As a result, there exists abundant data on medical research funding for the United States, which is advantageous in detecting the relationship between funding sources and medical research in a rigorous and detailed manner.

In short, the main research questions of this study are as follows: Is the origin of funding source—in particular, whether the funding originates from the government—related to the characteristics of the breast cancer research frames? If so, what are the characteristics and causes of that relationship?

## Materials and methods

### Data

We collected 48,448 abstracts of breast cancer research papers from 1975 to 2016 to analyze breast cancer studies. We used PubMed to search for the papers and their abstracts, as its database is representative of professional breast cancer knowledge. The specific search criteria are as follows: (1) The paper has an abstract, (2) The paper is from a journal published in the United States, (3) The date of publishing is between 1975 and 2016, (4) The funding sources are either the US government agencies or are not indicated (This criterion excludes those studies that were funded by non-US government agencies), (5) The title of the paper contains the term “breast cancer,” and (6) The Medical Subject Headings (MeSH) Major Topic of the paper is “breast neoplasms.” The criteria are combined as (1)–(4) and then either (5) or (6). We relied on the abstracts of the papers, because their compressed content provides sufficient and crucial information for extracting the research frames, and their relatively uniform amount of text is adequate for controlling noise stemming from the difference in length of text.

A more detailed descriptions of criterion (4) and funding source distinctions are as follows. The funding sources of the papers are categorized as <Government> and <Private>. The <Government> group consists of papers supported by the US government institutions (e.g., National Institute of Health and Center for Disease Control and Prevention). The <Private> group consists of papers not supported by the US government. Methods for identifying the two categories are as follows. Data from PubMed contain fields called “PT” (Publication Type) and “GR” (Grant Number). Information on the official research funds granted for each paper is contained here. The papers classified as <Government> are those with the words "United States" written on the “GR” field. The papers classified as <Private> do not include the words “Research Support” on “PT” and the words “United States” on “GR”. In some papers, “Research Support” appears in “PT”, while “United States” does not appear in “GR.” These papers are mainly supported by other governments (e.g., German government) or their specific sources of funding cannot be identified. They are excluded. Finally, it should also be noted that data were collected on April 12, 2017.

### Method

We built on the definition of frames offered by framing theory (e.g. Entman 1993) and defined a research frame as a conceptional structure undergirding research that separates and engages with a specific aspect of the phenomenon under study. We argued that research frames are expressed by the frequently appearing words and the unique network among them. The frequently appearing words indicate what the author of the study wants to highlight. However, words alone do not reveal the meaning and value of the object. As Saussure pointed out, the meaning and value of a word are produced through its relation with other words [29]. In short, the frequently appearing words and their networks indicate which objects the authors are trying to highlight and what meaning and value the authors are attaching to the words. These are valuable clues to tracking research frames.

We used the following three methods to extract the research frames from the paper abstracts and to analyze the relationship between funding and the research frames: (1) structural topic modeling (STM), (2) word network analysis, and (3) LASSO logistics regression. The reasons for the use of these three methods are as follows. STM captures the research framings and the differences in proportion of multiple frames depending on funding sources. Word network analysis identifies the main objects for each category (<Government> and <Private>) based on the relationship between words that cannot be captured directly by STM. Therefore, word network analysis would supplement the conclusions from STM based on other aspects of data. However, these two methods involve human interpretation, at least in part, in the process of drawing conclusions. We introduced a third method, LASSO logistic regression, to supplement these two methods, in a more objective manner. LASSO logistic regression selects important variables, in this case, words, for predicting the funding sources of each paper. The result of LASSO logistic regression could confirm the conclusion based on the previous methods.

#### Structural topic model

STM is used to extract multiple frames from the abstracts and to test the hypotheses on the relationship between funding sources and research frames. Topic modeling is a type of method, which infers “topics”—generally defined as probability distributions of words which are most likely to generate the given text—from a collection of documents and also the distribution of topics in each document [30, 31]. (The above definition of “topics” is based on the Latent Dirichlet Allocation (LDA) approach, one of the most widely used approaches in the field [32]. Our discussion of topic modeling’s basic features is largely based on LDA). Moreover, STM is a specific topic modeling method that allows researchers to estimate the relationship of topics to document metadata (e.g., publication date, author, journal category) in addition to inferring topics [33]. If we incorporate funding sources of each research article into our topic model using STM, we can estimate the relationship between a topic’s proportion and funding sources.

Two pieces of information can be easily obtained from the “topics”—which words are assigned a high probability and a cluster of such words. From this, we can infer the two factors mentioned above: the frequently appearing words and their networks. For example, let us assume that the following topic exists: [mammography– 0.06; breast– 0.05; cancer– 0.05; region– 0.04; screening– 0.03, difference– 0.03; attitude– 0.03; receive– 0.03; people– 0.02; rate—0.02]. This is a list of top ten words based on probability. If we observe this topic, we can assume that the word ‘mammography’ is an important object in the data; only when ‘mammography’ appears a lot can the word be given a high probability in a topic estimated from the data. Furthermore, from the set of words, we can see in what context and meaning these words are used, at least roughly. From the above set, we can infer that the text data focuses on the regional difference of people receiving mammography. The information reveals a framing that exists in the text data. To sum up, the high-probability words and their clusters, identified from these “topics,” constitute the frame.

Moreover, topic modeling assumes that a document is composed of multiple topics and estimates these multiple topics from a set of documents. This is a realistic assumption because a single document (in this case, a research paper abstract) can have multiple subjects and focuses. With this assumption, topic modeling not only estimates multiple topics but also topics’ proportions in each document, which enables us to determine the prevalent topic in each document. This result can be interpreted as the prevalence of a specific frame in a document.

What STM adds to regular topic modeling is that it enables the researcher to discover the relationship between topics and various metadata of the document. In this case, we estimate the relationship between funding sources and proportions of topics, controlling for the effects of time. If our structural topic model reports a significant difference in the proportions of topics and thus research frames depending on funding sources, then it can be interpreted as an indication that a relationship exists between funding sources and research frames. For example, if a frame that focuses on genes is more prevalent in studies supported by government institutions, then it implies that government funding is associated with molecularization.

#### Word network analysis

Word network analysis measures the relations of concepts, which is one aspect of frames, more directly than topic modeling. Although a cluster of words extracted from each topic provides some information regarding the relations between words, this is not a direct measure of the relations. Different words can have high probabilities at the same time in one topic, even though they are not connected by any criteria. This study constructs word networks using data from the abstracts based on the assumption that the co-occurrence of words in the same sentence means a connection. We divided the abstracts into two groups—those funded by the US government and those marked as not having a funding source—and constructed separate word networks for them. This facilitated the comparison of overall networks and the various network indices, for example, degree centrality and betweenness centrality.

#### LASSO logistic regression

LASSO logistic regression is employed to support our arguments in a more objective manner. The preceding methods have one common characteristic—they need human interpretation. A topic is a probability distribution. We interpret the distribution to extract meaning (as in the example topic above). Additionally, it is essential to interpret network indices (e.g., centrality) as having a high or low score because they are continuous values rather than discrete values. We construct a logistic regression model that takes the words in each abstract as explanatory variables and the funding source category as a response variable to support our argument. In this model, the number of explanatory variables reaches about 52% of the number of cases (abstracts), resulting in overfitting. LASSO logistic regression overcomes the overfitting problem and selects key variables [34, 35] at the same time. Using LASSO logistic regression, we extracted 2,719 words that were assumed to have a significant role in predicting the document’s funding source. Among these words, we chose the top thirty that had the strongest relationship with the document’s funding source in positive and negative directions, based on standardized coefficients. This result was used to identify the important objects in each funding source.

#### Pre-processing and statistical tools

Finally, we used R (a statistical programming language) and several packages in R for data preprocessing and analysis. For each of the analyses mentioned above, data preprocessing is slightly different. In the case of STM, the following operations were performed: switching all characters to lowercase, stemming, removing punctuations, removing stopwords, removing numbers, and removing words that appeared in just one document. Stemming reduces words to their word stem. The endings of several words are modified as a result of stemming because it turns various words that come from the same stem into the same form (e.g., disease, diseases ->diseas). Stopwords are the most commonly used words that do not have significant meanings (e.g., a, an, the). These operations for STM and tokenization were done using the stm package (version 1.3.3).

In the case of LASSO logistic regression, the following operations were performed: switching all characters to lowercase, stemming, removing punctuation, removing stopwords, removing words containing only numbers, and removing words that appeared in just one document. These operations for LASSO regression and tokenization were done using the tidyverse package (version 1.2.1), tidytext package (version 0.2.1), and SnowballC package (version 0.6.0). In the LASSO regression, we considered only which words appeared in each document and not how many times they appeared. This was to analyze how the presence of each word in the document itself affected the prediction of the funding sources. We used the glmnet package (version 2.0–18) in R to perform cross-validation and construct the LASSO logistic regression model.

For the word network analysis, seven operations were performed. To begin, the following preprocessing tasks were applied: switching all characters to lowercase, stemming, removing punctuation, removing stopwords, and removing words containing only numbers. On top of these, two more operations were added: (1) We deleted several words that were used only to classify the content of the abstracts. For example, if “Purpose:” or “Method:” appeared at the beginning of a sentence, then such words would not have a significant meaning, and they would only function to inform readers about the nature of the following text. Based on this reasoning, we deleted “aim:,” “purpose:,” “background:,” “backgrounds:,” “method:,” “methods:,” “result:,” “results:,” “conclusion:,” “conclusions:,” “objective:,” and “objectives:,” if the words appeared at the beginning of a sentence. (2) We deleted “breast,” “study,” and “studies” because they do not have significant meanings, considering that the data is from breast cancer research paper abstracts. These two preprocessing were introduced only in the word network analysis because STM and LASSO regression include analytical logic and processes to exclude the effects of these words, but word network analysis does not. These preprocessing tasks and tokenization were done using the tidyverse package (version 1.2.1), tidytext package (version 0.2.1), and SnowballC package (version 0.6.0).

## Results

### STM results

First, we extracted twenty topics from the abstracts using STM, considering the funding source and time (year) as covariates. We assumed that the funding source category and time would influence topic proportions in each abstract. We made a model based on the assumptions using the stm package (version 1.3.3) in R. Subsequently, we interpreted the topics with the help of two medical professionals (One is a family medicine specialist and the other is a researcher with a Ph.D. in preventive medicine) using twenty important words of each topic and five abstracts in which the topic was most prevalent.

The important words of each topic were selected on the basis of two criteria: ten words based on the highest probability and ten words based on the highest Frequency-Exclusivity (FREX) score. The FREX score is used to identify important words by taking into account both a word’s frequency under a topic and its exclusivity to that topic [36].

In addition, a unit of interest was also assigned to each topic, indicating the focus of the research. To each topic, we assigned one of the four levelsas a unit of interest—<Molecular level>, <Mixed>, <Individual level>, and <Group level>. <Molecular level> means topics dealing with the object and dynamics at the cellular level (e.g., DNA and cell receptor). <Mixed> refers to topics with both molecular objects and bigger objects, like patients, as the unit of interest (e.g., research into the effects of molecular objects on patient survival rate). <Individual level> denotes topics primarily dealing with individual patients as a unit of interest (e.g., research into the effects of various treatments on patients). <Group level> means topics dealing with group-level objects (e.g., race and cohort).

We treated the interpretations and the units of interests as features of the breast cancer research frame because these were based on significant words and their relationships, which are crucial aspects of a research frame. Table 1 gives an example of interpretations given to topic 03.

**Table 1:** Example of topic interpretation (topic 03).

Titles of papers that have the highest proposition of the topic	Important words of the topic	Unit of interest	Interpretation
Breast cancer incidence and mortality trends in Missouri	Highest probability: women, cancer, breast, screen, age, year, use, rate, mammographi, among	**Group level**	**Studies of macro factors that influence breast cancer incidence rate, such as race, geographic location, and insurance.**
Declining mammography screening in a state Medicaid Fee-for-Service program: 1999–2008			
Breast and cervical cancer screening: impact of health insurance status, ethnicity, and nativity of Latinas			
	FREX: black, medicar, seer, racial, non-hispan, raceethn, ses, white, hispan, racialethn		
Mammography use helps to explain differences in breast cancer stage at diagnosis between older black and white women			
Racial trends in age-specific breast cancer mortality rates in US women			

**Fig 1:**
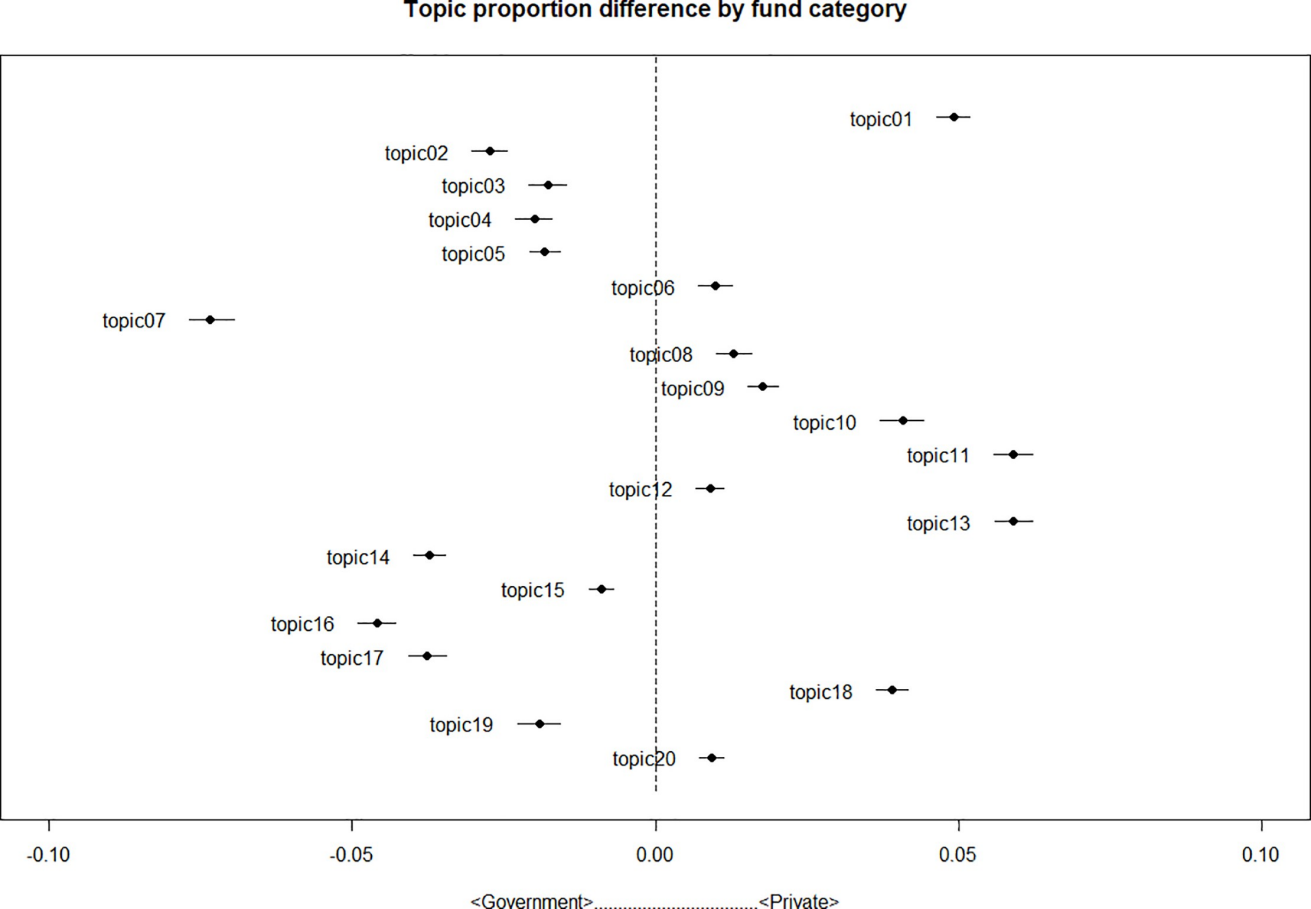
Topic proportion difference by funding source.

As mentioned earlier, the key function of STM in this paper is to estimate topic proportion differences that are associated with changes in the covariates. In this case, we used the funding source category and time as covariates and subsequently estimated the relationship between funding sources and the topic proportions. Fig 1 shows each topic’s difference in proportions by funding sources. In the figure, the dots represent the estimates of average proportion differences for each topic by funding sources, and the whiskers represent the 95% confidence intervals for each estimate. For example, topic 01 is shown to have a larger share in papers of the <Private> category, whereas topic 07 is shown to have a larger share in papers of the <Government> category.

Table 2 summarizes each topic’s interpretation, unit of interest, and the category where the topic’s proportion is higher. Table 3 presents a summary of the topics’ focus and proportion difference by funding sources.

**Table 2:** Topic’s interpretation, unit of interest, and prevalent category.

No.	Interpretation	Unit of interest	Category where the topic’s proportion is higher
1	Review research on the management and treatment guidelines of breast cancer	Mixed	<Private>
2	Pathological features of cancer cells	Molecular Level	<Government>
3	Studies of macro factors (such as race and geographic location) that influence breast cancer incidence rate	Group Level	<Government>
4	Research on gene sequences that influence breast cancer incidence	Molecular Level	<Government>
5	Research on targeted drugs or methods	Mixed	<Government>
6	Research on radiation therapy	Individual Level	<Private>
7	Research on transcription factors and suppressors related to metastasizing	Molecular Level	<Government>
8	Results of clinical trials on adjuvant chemotherapy	Individual Level	<Private>
9	Research on a cell’s hormone receptors and triple-negative breast cancer	Mixed	<Private>
10	Diagnosis methods of breast cancer using various visual images and biopsy	Individual Level	<Private>
11	Various types of tumor and related diseases in the breast	Mixed	<Private>
12	Research on hormone therapy (e.g., effect of aromatase inhibitor)	Individual Level	<Private>
13	Various surgical treatments, including breast reconstruction	Individual Level	<Private>
14	Mechanism of the breast cancer cell from the perspective of molecular biology	Molecular Level	<Government>
15	Research on a breast cancer cell using various image techniques/results of animal experiments	Molecular level	<Government>
16	Factors that influence breast cancer cell growth	Molecular level	<Government>
17	Dietary habits and breast cancer (mainly cohort studies)	Group Level	<Government>
18	Research on lymph nodes	Mixed	<Private>
19	Psychosocial factors that influence a person’s coping capacity for the stress caused by breast cancer (e.g., social network factor)	Individual level	<Government>
20	Research on a cell’s receptors and related antibodies	Molecular level	<Private>

**Table 3:** Topics’ focus and proportion difference by funding sources.

Focus of topic	Number of topics whose proportion is higher in <Government>	Number of topics whose proportion is higher in <Private>
Molecular level	6 (60%)	1 (10%)
Mixed	1 (10%)	4 (40%)
Individual level	1 (10%)	5 (50%)
Group level	2 (20%)	0 (0%)

The STM results reveal three interesting points. First, studies funded by the government are more likely to feature topics that have molecular objects as their unit of interest. Second, studies conducted in the private sector are more likely to include topics that focus on patients. Most topics belonging to <Individual level> mainly deal with patients. For example, topic 08 (results of clinical trials on adjuvant chemotherapy) and topic 13 (various surgical treatments, including breast reconstruction) are specific issues on patient treatments. Topics belonging to <Mixed> also pay attention to patients. That is, even when they deal with molecular level objects, like cancer cells, they deal with those objects in relation to patients. For example, topic 5 focuses on targeted drugs. It pays attention to the molecular level mechanism; however, the main purpose of the focus is curing patients. According to Table 3, <Mixed> and <Individual level> are more prevalent in the <Private> category. Third, two topics (3 and 17) take group-level objects such as race and cohorts as the unit of interest, which are relevant concepts when dealing with populations, not individuals. The proportion of the topics tends to increase if a study is funded by the government. Many studies have pointed out that governments are interested in populations, rather than in individuals [37]. The findings herein are consistent with the findings of those previous studies.

These three findings demonstrate that government funds are associated with a focus on molecular objects or the population as the unit of interest, and private sector conditions are associated with frames that focus on individual patients. Previous research has generally given equal weight to the roles of governmental and private organizations in molecularization [11]. However, the STM results show that the government plays a far more active role in molecularization. This conclusion is further strengthened by the word network analysis and LASSO logistic regression that follows.

### Word network results

As stated previously, STM cannot directly show the relations between words, which are important information in identifying frames. Even though topic modeling extracts a cluster of significant words (e.g., ten highest-ranking FREX words and ten highest probability words in Table 1), the cluster cannot guarantee the connection among the words. Therefore, word network analysis is also used in this research. We divided the data into two categories based on the source of funding—18,842 papers in the <Government> category and 29,606 papers in the <Private> category—and built word networks in each collection. We assumed that the co-occurrence of words in the same sentence means that there is a connection between the terms. For example, consider a sentence that reads “gene affects cancer cell growth.” In this case, “cancer” and “gene” can be considered as being connected. We used igraph (version 1.2.4.1), widyr (version 0.1.1), tidytext (version 0.2.1), and tidyverse (version 1.2.1) packages in R to build each word network.

The constructed word networks give various information. First, we analyzed which words are connected to “cancer” and “tumor” as well as the number of connections; this is because connections with the maximum frequency identify the concepts that are considered important in relation to cancer and tumor in each category. Differences between the two groups could mean that different frames are dealing with cancer and tumor. Second, we measured the centrality score of words in each network and ranked the words. We expected the centrality score to reveal the important objects in each category.

The analyses revealed that words indicating molecular objects are connected with “cancer” and “tumor” more frequently than other words in the studies funded by the government. They also show higher centrality scores than other words in a word network of these studies, which means that the words indicating molecular objects occupy central positions in the word network. On the contrary, words indicating patients or specific treatments are more frequently connected with “cancer” and “tumor” and show higher centrality scores in a word network of the studies in the private sector. The concrete results are as follows. Table 4 shows the frequently connected words (the top twenty list), with “tumor” and “cancer” in each network.

**Table 4:** High-rank words (criteria: Weight) connected to “tumor” and “cancer”.

Connection with "tumor"				Connection with "cancer"			
Studies funded by the government	Weight	Studies in the private sector	Weight	Studies funded by the government	Weight	Studies in the private sector	Weight
cell	6301	patient	5953	cell	11329	patient	18516
cancer	5524	cancer	4717	women	10102	women	10210
express	3387	cell	4009	risk	9795	risk	7527
patient	2809	size	2988	patient	8152	treatment	7277
growth	2493	posit	2453	tumor	5524	cell	6120
receptor	1911	node	2352	express	5143	therapi	5022
human	1799	primari	2324	increas	4394	tumor	4717
posit	1620	express	2139	human	4342	stage	4490
gene	1615	receptor	2055	treatment	4123	clinic	4206
activ	1517	carcinoma	1810	factor	3591	increas	3945
increas	1514	grade	1724	effect	3515	effect	3776
primari	1511	treatment	1655	result	3489	diseas	3607
treatment	1404	neg	1631	gene	3447	factor	3496
mice	1378	lymph	1590	activ	3402	result	3447
result	1313	statu	1505	receptor	3227	develop	3435
tissu	1299	factor	1483	ag	3152	posit	3404
model	1298	invas	1455	suggest	3085	detect	3401
estrogen	1297	clinic	1421	therapi	3051	screen	3394
er	1288	stage	1419	data	3009	express	3267
effect	1267	therapi	1400	posit	2927	metastat	3248

The word most frequently connected to “cancer” and “tumor” and unique words in each category show that research focuses are quite different between the two categories. First, the word most frequently connected to “cancer” and “tumor” is “cell” in the studies funded by the government and “patient” in studies in the private sector. This means that cancer and tumor are dealt with mainly in relation to cells in government-funded studies and patients in studies from the private sector. This implies a clear difference in perspective between the two categories.

Second, the unique words from the top twenty list in each category (words in shaded cells in Table 4) also show differences in framings. Let us start with the set of words associated with “tumor.” While the words that can be found in the top 20 lists of both categories (<Government> and <Private>) indicate commonly important words, the unique words in each category show the unique focus of each category. From government-funded studies, “growth,” “human,” “gene,” “activ,” “increas,” “mice,” “result,” “tissu,” “model,” “estrogen,” “er,” and “effect” are the unique set of words and they indicate the molecular objects (gene, estrogen, er [estrogen receptor]) or the state change of the molecular objects (growth, activ) or the research process (human, mice, result, model). On the other hand, from the studies in the private sector, quite different unique words are identified, which are “size,” “node,” “carcinoma,” “grade,” “neg,” “lymph,” “statu,” “factor,” “invas,” “clinic,” “stage,” and “therapi.” The set of words is mainly about patient treatment, because the words are directly indicating clinical treatment (clinic, therapi) or information that is important for patient prognosis (size, neg, invas, stage) or the body location where the information is identified (node, lymph). This discrepancy indicates that government-funded research has framings focusing on molecular objects, whereas the studies in the private sector have patient-oriented framings.

A similar tendency is also identified from the word sets connected to “cancer.” From government-funded studies, “human,” “gene,”, “active,”, “receptor,” “ag,” “suggest,” and “data” are identified as unique words, whereas in the studies in the private sector, “stage,” “clinic,” “diseas,”, “develop,” “detect,” “screen,” and “metasta” constitute the set of unique words. In the set from government-funded studies, we can see the words indicating molecular object (gene, receptor), change of state of the molecular object (active), and the research process (human, data). In the set from private sector research, we can see the words about treatment (clinic, diseas) and information that is important for patient prognosis (stage, metasta).

**Fig 2:**
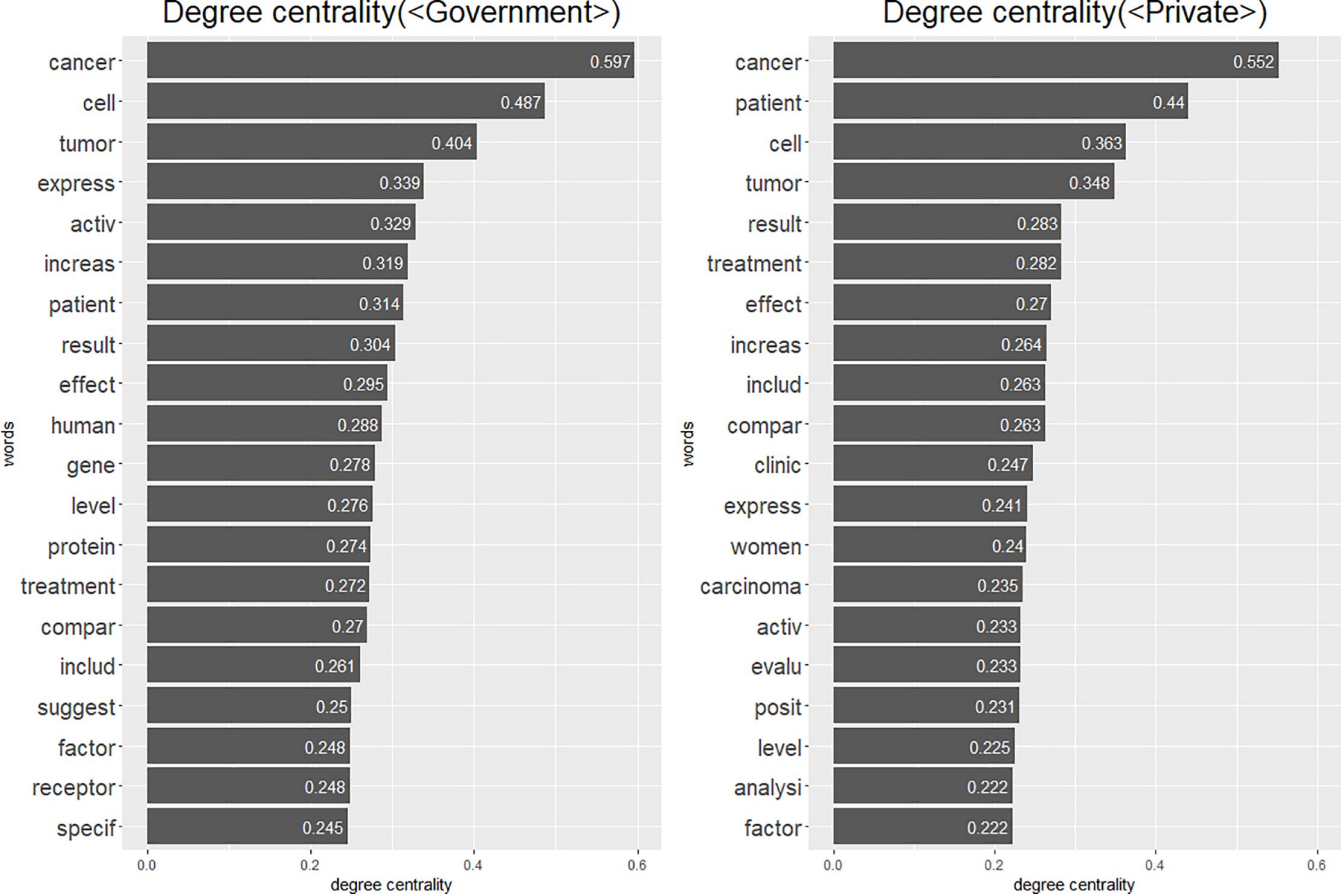
Degree centrality.

The results of the centrality indices are consistent with the result of frequently connected words. Various centrality indices can be used to identify the important nodes (in this case, words) in a network. Compared to simple frequency, centrality indices provide more nuanced information that helps determine which objects are important. This is because centrality indices capture a node’s importance by considering the node’s relationship with other nodes. We use the igraph package (version 1.2.4.1) in R for the network analysis. First, we calculated the degree centrality of words from each word network and produced the list of the top twenty words from each network. Fig 2 shows the result.

In the <Government> category, we find that the word “cell” has the highest position in terms of degree centrality, after excluding obvious words (cancer and tumor). Additionally, the molecular objects like “gene,” “protein,” and “receptor” only appear in the list of <Government> category. On the other hand, the word “patient” has the highest position in the <Private> category, after excluding obvious words (cancer and tumor). The word “clinic,” which is deeply related to the patient treatment, only appears in the word list of <Private> category. These results are consistent with the results of words’ connections with “cancer” and “tumor.” That is, these results indicate that studies in the <Government> category tend to have framings focusing on molecular objects, whereas the studies in the <Private> category are likely to have patient-oriented framings.

**Fig 3:**
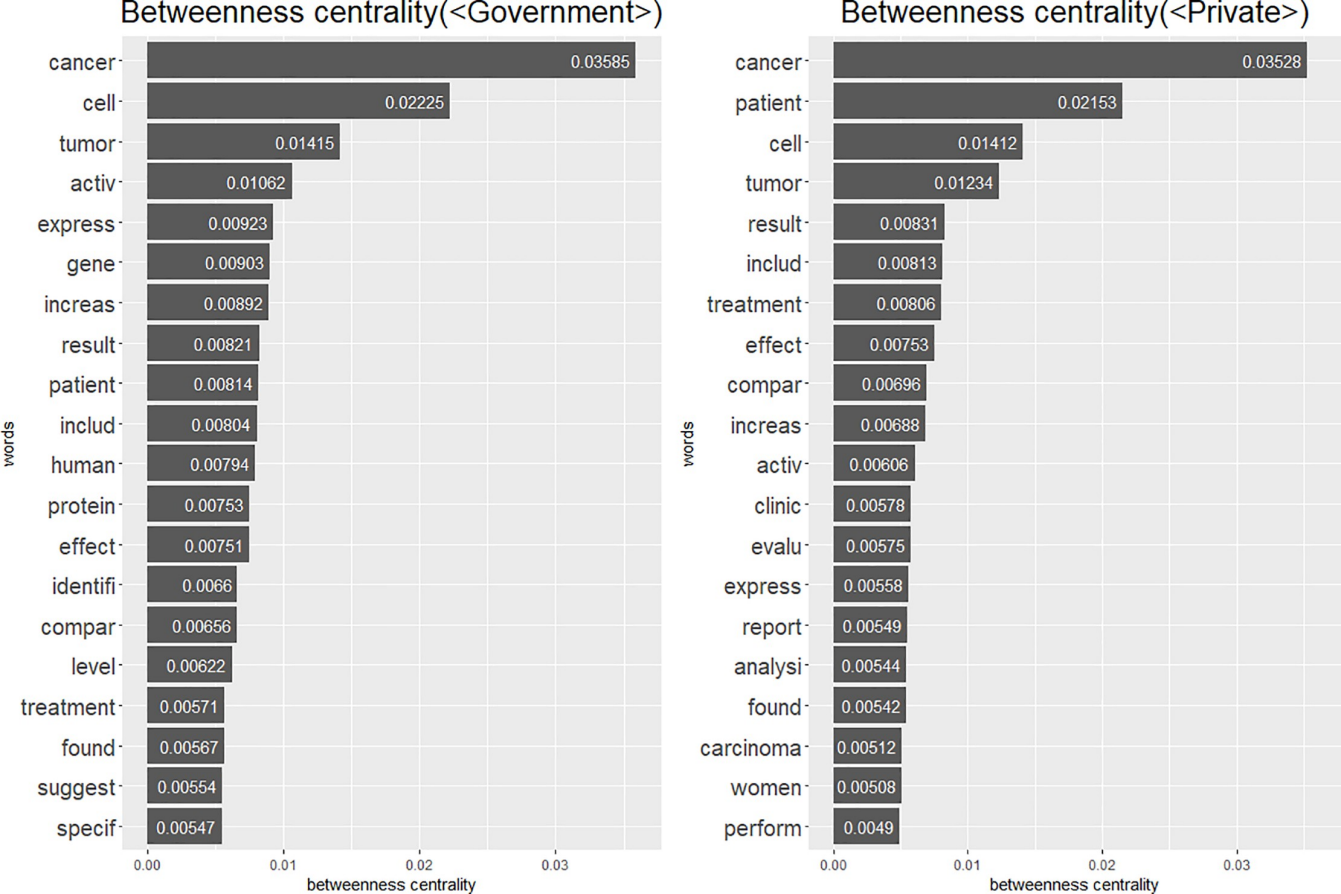
Betweenness centrality.

However, degree centrality only considers direct links of nodes. This means that the words that are frequently connected with only a limited group of words in a network can have a high degree centrality score. Therefore, a word’s high degree centrality score cannot guarantee its importance in an entire network. Alternatively, the betweenness centrality measures a node’s centrality by considering the intermediary role of the node [38, 39]. The high score of betweenness centrality means that the node frequently appears on the shortest path between the other node pairs. This index is especially useful for detecting central positions when the nodes form separated subgroups in a network, in which the intermediate role becomes important. We utilized betweenness centrality to overcome degree centrality’s limitations and measure nodes’ centrality considering the whole network structure, not just the direct connection of the nodes. Certainly, betweenness centrality is not the only alternative to deal with degree centrality’s limitations. For example, closeness centrality measures global centrality based on the distance between nodes [38, 39]. However, considering the characteristics of our data that it is highly likely that multiple clusters of words exist because of various subjects of research, we concluded that betweenness centrality is more suitable than closeness centrality. The betweenness centrality index obtained is consistent with the previous results (Fig 3).

The betweenness centrality indices also show that studies in the <Government> category consider cells to be important, whereas studies in the <Private> category focus on patients. The words “cell” and “patient” still appear as the most central words (after excluding the obvious words, “cancer” and “tumor”) in the <Government> category and the <Private> category, respectively; “gene” and “protein” only appear in the <Government> category list; and “clinic” only appears in the <Private> category list.

These results mean that, even though the same disease is studied, there can be differences in the units of interest and relations between concepts depending on the source of funding. The studies supported by the government are more likely to investigate breast cancer from a perspective that focuses on changes in the state within the cell, whereas other research papers are more likely to focus on patient treatment and prognosis. The results show the differences in the frame between government-supported research and other research.

### LASSO logistic regression

LASSO logistic regression is used to support the previous results in a more statistical manner. STM and word network analyses need human interpretation. STM needs topic interpretation and centrality indices need to be interpreted as high or low because they are continuous rather than discrete values.

We construct a logistic regression model that predicts a study’s funding category (<Government> and <Private>) based on the words appearing in the study. That is, in this model, the explanatory variables are words that appear in the data, and they are given dichotomous values depending on whether the word exists in that abstract (1 –exist, 0 –not exist). As we mentioned earlier, the goal here is to analyze how the presence of each word in the document affects the prediction of the funding sources of that document. If we can extract significant words that contribute toward predicting a study’s funding source, then those extracted words can be interpreted as important objects or subjects in each category.

However, this model has too many explanatory variables because there were 25,397 distinct words after shortening the list with preprocessing. This represents about 52% of the number of cases (48,448), which causes overfitting and decrease the model’s value. LASSO regression resolves the problem of overfitting and selects the important terms in each category.

A LASSO regression model reduces the absolute values of coefficients systematically to enhance its predictive ability. The reduced coefficients contribute to increasing the predictive ability of a model because they decrease the model variance. LASSO regression is a type of penalized model [35]. An ordinary logistic regression method estimates coefficients of a model to maximize the log-likelihood of the given data (see formula below).

$$\frac{1}{N}Log\left(\prod_{i:y_{i}=1}p(X_{i})\prod_{j:y_{j}=0}\left(1-p(X_{j})\right)\right)$$

In comparison, a LASSO logistic regression model estimates the coefficients of the model to maximize the value of the formula below. Even though a set of coefficients produces maximum likelihood, if the absolute values of the coefficients are too high, they cannot guarantee the maximum value of the formula. In other words, the second part of the formula works as a shrinkage penalty. As the tuning parameter (lambda, λ) in the formula increases, the impact of the penalty also increases and the coefficients of the model approach zero. During this process, LASSO regression also selects the key variables because that makes the coefficients of unimportant variables approach zero before the coefficients of significant variables [34, 35].

$$\frac{1}{N}Log\left(\prod_{i:y_{i}=1}p(X_{i})\prod_{j:y_{j}=0}\left(1-p(X_{j})\right)\right)-\lambda \sum_{k=1}^{P}\left|\beta_{k}\right|$$

**Fig 4:**
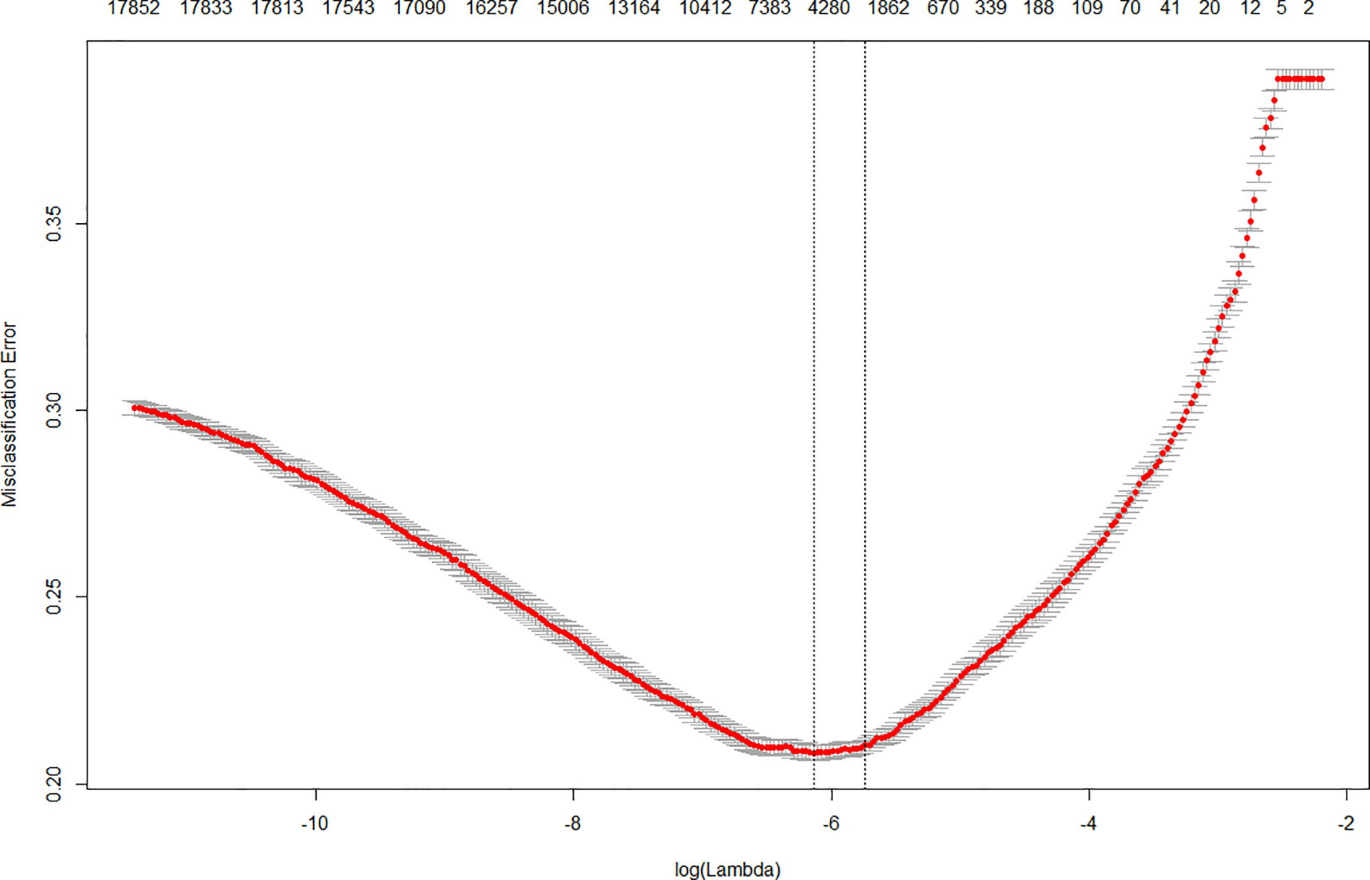
Misclassification error.

Therefore, the analyst needs to choose an appropriate value of lambda as it determines the shape of the model and the number of selected variables. Normally, the lambda value is determined based on the model’s prediction ability, calculated through cross-validation. Afterward, the analyst chooses the lambda for the model according to a criterion, such as choosing a lambda value that minimizes the model’s prediction error. We explored our lambda values by using a ten-fold cross-validation with the misclassification error rate as an indicator of model performance. Instead of choosing the lambda value of minimum error, we determined the lambda value of our LASSO logistic regression model based on the “one-standard-error” rule, which chooses the largest lambda value within one standard error that minimizes the model’s mean prediction error. This rule takes into account the fact that the mean prediction error of a model with specific lambda value is also estimated with error [40] and allows the researcher to “choose the simplest model whose accuracy is comparable with the best model” [41]. This is a suitable criterion for our model because our objective is to select small groups of significant variables and make a powerful model. The lambda value, which minimizes the prediction error, was about 0.00216, and the lambda value based on the "one-standard-error" rule was about 0.00322. (Fig 4 shows the relationship between misclassification error rate and lambda value.) We formed a LASSO logistic regression model based on the latter value. We used the glmnet package (version 2.0–18) in R to perform cross-validation and construct the LASSO logistic regression model.

The LASSO logistic regression extracted 2,719 words from 25,397 candidates considered important in predicting the funding sources. The model also estimated the coefficients of these words. These 2,719 variables extracted are more important in predicting the funding category than those that have become zero earlier. However, 2,719 variables are too numerous to extract meaningful information from them. To find the explanatory variables (words) that have the strongest relationships with the response variable (funding category), we calculated the standardized coefficients of the explanatory variables. The standardized coefficients are calculated by the method suggested by Menard and Agresti [42], in which a standardized coefficient of an explanatory variable is the product of an unstandardized coefficient and a standard deviation of the variable.

$$\beta_{a}^{standardized}=\beta_{a}^{unstandardized}\times \partial_{a}$$

The reasons for using standardized regression coefficients instead of non-standardized ones are as follows. Since non-standardized coefficients of variables do not take into account the variance of the variables, we cannot determine the importance of any variable just by the absolute value size of the non-standardized coefficients. For example, let’s assume the word ‘Atew’ (a made-up word for example) appears only in three of the 48,448 documents, all of which received funds from the government. In this case, the regression coefficients of the word ‘Atew’ would be very high. However, it is hard to say that the word ‘Atew’ is important since it rarely appears. Even if the word has a high regression coefficient, in this case, it is reasonable to say that the word has little influence because the value of the variable (that is, the appearance of the word in each document) is zero in most cases.

However, if we calculate the standardized logistic regression coefficients in the manner presented by Agresti and Menard, it will tell us how much the value of logit Y changes when the explanatory variable varies by one standard deviation. These standardized regression coefficients are more consistent with our analysis objectives. The lower frequency word (“Atew”) has very small variance because most values are zero (In this case, the mean of the variable is almost zero and the variance is also close to zero). Although the regression coefficient assigned to the lower frequency variable is large when not standardized, it becomes small when standardized. Therefore, we can evaluate each words’ importance accurately based on the standardized coefficient. In short, we have introduced standardized logistic regression coefficients to identify the important words in each category by taking into account the variances of each variable.

Based on standardized coefficients, thirty variables each with the highest and the lowest values were identified. See Table 5 for the results. The first thirty variables contribute most to a paper being classified as <Government> category and the second thirty variables contribute most in the opposite direction. We interpreted these as the most important words in each category because the appearance of these words in the paper’s abstract greatly increases the probability of the paper being classified into each category.

**Table 5:** Top standardized coefficients of the model.

positive top 30		negative top 30	
word	st.coef	word	st.coef
cell	0.139364755	patient	-0.15909593
model	0.110949997	aim	-0.157242413
confid	0.099055889	tumour	-0.154677616
cancer	0.093272847	epirubicin	-0.089756573
optic	0.089170934	literatur	-0.072798158
consortium	0.088136082	mtt	-0.06927753
receiv	0.079648671	retrospect	-0.068800323
mice	0.078649754	review	-0.067158606
human	0.078303703	complic	-0.059448332
enrol	0.075670692	radiotherapi	-0.057253076
adjust	0.074308389	flap	-0.055822645
mediat	0.072439714	carcinoma	-0.053531896
measur	0.071372035	consecut	-0.051143841
vivo	0.067774469	meta	-0.049526264
women	0.067716787	woman	-0.049134888
suggest	0.065751423	needl	-0.047191287
medicar	0.065477859	wilei	-0.045474514
particip	0.065447095	prognost	-0.045255925
cohort	0.060583469	articl	-0.044671359
telephon	0.059420701	investig	-0.044244178
baselin	0.059298868	grade	-0.044235874
gene	0.05636647	perform	-0.044213184
purpos	0.055491833	excis	-0.042265452
randomli	0.054134345	manag	-0.041605671
regul	0.053822285	histolog	-0.041532234
shanghai	0.053380027	lesion	-0.041056099
associ	0.052359973	hospit	-0.04102179
survivor	0.052118128	italian	-0.039853974
result	0.052111729	world	-0.038452841
angel	0.051892966	centr	-0.037849038

The LASSO logistic regression model offers results that are consistent with the previous analyses (STM and network analysis). The word that has the strongest positive relationship to the study being classified into the <government> category is identified as “cell.” The word “gene” is also found in the positive top thirty standardized coefficients list. On the other hand, in the negative top thirty standardized coefficients list, “patient” is ranked first. This suggests that the appearance of the word “patient” has a strong negative relationship to a research paper being classified into the <government> category—in other words, it has a strong positive relationship to being classified into the <private> category. In addition, numerous words related to the actual treatment of patients are found on the negative top thirty list. The words indicating treatment methods (radiotherapi, epirubicin), the words related to surgery (flap, needl, excis), and the words related to patient condition and prognosis (complic, prognost) are examples. These results suggest that molecular objects are important in the research frame of government-funded studies, whereas patients and patient-related objects are important in the research frame of studies in the private sector.

Further, words related to the population are also found to have strong positive relationships with the response variable. The word “cohort” in the positive list is a good example, as a cohort means a group of subjects, not an individual. The contrast between “woman” and “women” is also an interesting result that shows differences in units of interests between the government and the private sector. While “woman” is in the negative top thirty list, “women” is in the positive top thirty list. These results indicate that the government tends to focus on the population or group level, whereas the private sector tends to focus on the individual patient level.

## Discussion

The results of this study show that the focus unit of research is related to the source of funding. If research is funded by government agencies, then the focus units of the research will likely be micro-objects, such as cancer cells and genes—which we refer to as “molecular objects”—or macro-objects, such as a cohort and specific groups of patients. Conversely, if research is done in the private sector, the focus units of research will more likely be breast cancer patients. These findings are consistent across the three analyses.

We propose that this phenomenon is due to the different interests of government institutions and the private sector. Governments have an interest in managing the population’s health, rather than that of a specific individual and in controlling medical expenses. This is because these are important ways of increasing the national budget and making the country more powerful [37, 43]. As a result, they focus on patient groups rather than on individual patients.

Devoting attention to “molecular objects” enables the following: (1) creation of knowledge that can be applied to the entire population, (2) screening of the population to identify risk groups and to concentrate medical resources on those groups. Knowledge of molecular objects is universal and not limited to specific patients, which is appropriate for the government. Further, knowledge of molecular objects is necessary when screening for cancer. Screening is for those members of the population who do not show any symptoms. At this stage, even a slight change at the molecular level can identify a person at risk. Screening is more likely to succeed if we understand the pre-cancer states well at the molecular level (cervical cancer screening is a good example). Successful screening means that the government can use medical resources efficiently and save on the national health insurance budget because the treatment cost of early-stage cancer or a pre-cancerous state is much lower than that of late-stage cancer [44, 45].

Conversely, the private sector and private organizations, including pharmaceutical companies and patient groups, have an interest in improving the outcomes of cancer treatments for individual patients. This is because such improvements can lead to economic benefits or the fulfillment of humanitarian goals. Cells do not pay or scream. Patients do. Therefore, research in the private sector pays more attention to patients and deals with cancer cells in relation to specific treatments.

The findings of this study improve our understanding of molecularization, which has been identified by various researchers. Most previous studies considered molecularization as a universal phenomenon and assumed that all important actors (the government, the pharmaceutical industry, and consumers) contribute to the phenomenon. However, these actors have different positions and perspectives on the molecularization process, and it is essential to consider these different stances when dealing with the effects of the process of molecularization.

Furthermore, this research enhances our understanding of medical research production. Our research demonstrates that funding sources relate not only to the content or conclusion of research but also to its very framing. Thus, medical research funding has a more fundamental relationship with research than depicted by existing literature, in that funding can affect which types of questions are asked in the first place and how those questions are addressed.
